# Novel Isolate of *Halobacteriovorax* Capable of Killing Multi-Drug-Resistant *Escherichia coli* and *Salmonella*

**DOI:** 10.3390/antibiotics14111133

**Published:** 2025-11-08

**Authors:** Stefania Di Lullo, Silvia Pieralisi, Giulia Talevi, Gabriele Angelico, Elena Rocchegiani, Francesca Leoni, Maira Napoleoni, Diego Maiolatesi, Francesca Barchiesi, Sara Nardi, Annalisa Petruzzelli, Claudia Gabucci, Angela Conti, Gianluigi Cardinali, Donatella Ottaviani

**Affiliations:** 1 Istituto Zooprofilattico Sperimentale dell’Umbria e delle Marche “Togo Rosati”, 06132 Perugia, Italy; s.dilullo@izsum.it (S.D.L.); s.pieralisi@izsum.it (S.P.); m.napoleoni@izsum.it (M.N.); c.gabucci@izsum.it (C.G.); d.ottaviani@izsum.it (D.O.); 2 Department of Pharmaceutical Sciences, University of Perugia, 06132 Perugia, Italy

**Keywords:** *Bdellovibrio* and like organisms (BALOs), *Halobacteriovorax*, *Salmonella* sp., *E. coli*, Antimicrobial-Resistant (AMR) pathogens, biocontrol agents

## Abstract

**Background/Objectives:** Due to the rising problem of antimicrobial resistance, there is increasing attention in the scientific community towards alternative approaches to combat Antimicrobial-Resistant (AMR) pathogens that do not involve the use of antibiotics. In this regard, the European Medicines Agency (EMA) and the European Food Safety Authority (EFSA) have promoted experimentation with predatory bacteria to fight antibiotic resistance. With the aim of identifying predatory bacteria suitable for the control of antibiotic-resistant bacteria, in this work we isolated a strain of *Halobacteriovorax* from an estuarine aquatic environment using a CTX-M-producing *E. coli* strain as prey and characterized it with respect to optimal physico-chemical parameters for growth and predation. Furthermore, we studied its predatory capacity against other *E. coli* strains and Multi-Drug-Resistant (MDR) *Salmonella*. Finally, we conducted challenge experiments to evaluate the growth of predator and prey over time. **Methods:** The *Halobacteriovorax* strain, designated HE7, was identified by 16S rRNA analysis. To isolate *Halobacteriovorax* and to evaluate its predatory ability towards different preys, the double-layer agar plating technique was applied. **Results:** HE7 showed in vitro predatory activity against all MDR strains of *E. coli* and *Salmonella* tested. In the 10^7^ predator/10^3^ prey and 10^7^ predator/10^7^ prey challenges, HE7 after 6 h achieved the total killing and a reduction of about 6 logs in the prey, respectively, maintaining this effect for up to 24 h. **Conclusions:** The results of this study highlight that HE7, but more generally *Halobacteriovorax*, could find application both alone and in an integrated context of antimicrobial strategies as an alternative to antibiotics.

## 1. Introduction

The excessive and often inappropriate use of antibiotics in medicine, agriculture and other activities has facilitated the selection of Antimicrobial-Resistant (AMR) pathogens, many of which are Multi-Drug-Resistant (MDR) [1,2]. The World Health Organization published the list of priority pathogens that have developed high levels of resistance worldwide and that constitute a danger to human health to which urgent responses must be found, particularly Gram-negative bacteria, also suggesting the development of new containment strategies [3,4]. Extended-spectrum cephalosporins and penicillins are important beta-lactams for the treatment and prevention of infections caused by Gram-negative pathogens but, as a result of their extensive use, extended-spectrum β-lactamase (ESBL) enzymes have emerged that can hydrolyze and confer resistance to these antimicrobials [5]. CTX-M (*bla*CTX-M genes), which originate from β-lactamase genes of environmental bacteria, have emerged as the predominant ESBL types since the new millennium [6]. Mobile genetic elements, such as transposons and integrons of environmental bacteria in water and soil, accelerate the transfer of *bla*CTX-M genes to animals and humans [2]. Over the last two decades, human intestinal transmission of CTX-M-producing *Enterobacteriaceae* has been increasing globally in both community and healthcare settings [7,8]. It is therefore urgent to search for new biological weapons capable of countering MDR bacteria and especially those that are CTX-M-producing. In this context, the use of bacterial predators as living antibiotics is yielding promising results, and, although more research is required to demonstrate their efficacy in vivo, they represent a serious alternative to be considered [2,9,10,11]. The European Medicines Agency (EMA) and the European Food Safety Authority (EFSA) have also promoted experimentation with predatory bacteria to fight antibiotic resistance [12,13]. Among predatory bacteria, *Bdellovibrio* and like organisms (BALOs), Gram-negative aerobic bacteria, selectively prey on other Gram-negative bacteria, making them a promising strategy to combat pathogens, including MDR pathogens [14,15]. Their use as living antibiotics is very promising due to some intrinsic characteristics of these predatory bacteria: a broad spectrum of activity, the absence of AMR genes that confer resistance to their prey, high penetration into biofilms and the apparent absence of specific resistance to them [9,10,11]. The taxonomic classification of BALOs is constantly evolving and they have now been included within two different classes, *Alphaproteobacteria* within the genus *Micavibrio* and *Deltaproteobacteria* within five families, *Bdellovibrionaceae*, *Peredibacteraceae*, *Bacteriovoracaceae*, *Pseudobacteriovoracaceae* and *Halobacteriovoraceae* [16,17,18]. However, until a few years ago halophilic species were included in the *Bacteriovoracaceae* family and identified in the genus *Bacteriovorax* [19,20,21], which was later reclassified as *Halobacteriovorax* [16]. Currently, the *Bacteriovoracaceae* family includes terrestrial species, while *Halobacterivoraceae* are ubiquitously present in saltwater environments, primarily seas and oceans, but also in low-salinity estuaries and high-salinity ponds [17]. The identification of BALOs has always been based mainly on the 16S rRNA gene sequence [19,20] but, considering recent taxonomic revision, to discriminate between *Bacteriovorax* and *Halobacteriovorax*, sodium chloride tolerance during growth must also be evaluated [16,17,18]. In fact, the growth of *Bacteriovorax*, unlike that of *Halobacteriovorax*, is inhibited by salt concentrations above 5 ppt [18]. To date, to the best of our knowledge, the studies regarding the use of BALOs in the fight against AMR bacteria have been focused on tests in vitro using the species *Bdellovibrio bacteriovorus*, either alone or in combination with classic antimicrobials or other novel modalities, such as bacteriophages [14,15,22,23,24]. In the marine and brackish water environment our group has previously isolated *Halobacteriovorax* strains with high predatory efficiency against a wide range of Gram-negative pathogens, halophilic, such as vibrios, but also non-halophilic, such as *E. coli* and *Salmonella* [25,26]. Identifying potential biocontrol agents such as *Halobacteriovorax* against AMR pathogens could provide valuable alternatives to conventional antimicrobials. This study aims to obtain preliminary data on the potential of *Halobacteriovorax* to prey on AMR *E. coli* and *Salmonella*. To identify predatory bacteria suitable for this scope in this work we isolated a strain of *Halobacteriovorax* from an estuarine aquatic environment using a CTX-M-producing *E. coli* strain as prey and characterized it with respect to optimal physico-chemical parameters for growth and predation. Furthermore, we studied its predatory capacity against other *E. coli* strains and MDR *Salmonella*. Finally, we conducted challenge experiments with different predator/prey concentrations to evaluate the efficacy of *Halobacteriovorax* in reducing prey levels over time.

## 2. Results

### 2.1. Halobacteriovorax Against E7 Detection

Out of two water samples analyzed, one was positive for presumptive *Halobacteriovorax* against E7. After 24 h, the plaques on the primary prey became visible, with a diameter of about 3 mm, and maintained approximately the same number and diameter until the end of incubation.

### 2.2. Halobacteriovorax Molecular Identification and Sequencing Analysis

**Figure 1:**
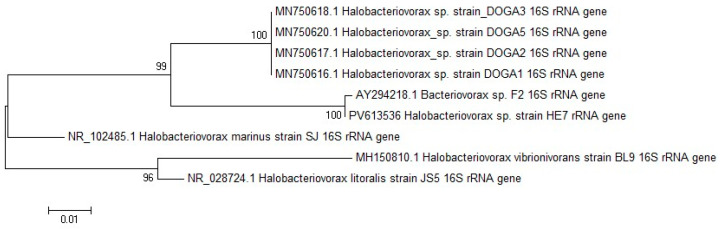
Maximum-likelihood phylogenetic tree, based on 16S rRNA gene sequence comparisons, showing the position of strain HE7 and related type strains. Numbers at branch nodes are bootstrap values (per 1000 trials).

All plaques for the positive sample were confirmed by PCR as *Halobacteriovorax*. Furthermore, the sequence obtained by analysis of the 16S rRNA 700 pb PCR fragment was named HE7. The 16S rRNA gene sequence of HE7 was deposited in GenBank under accession number PV613536. The maximum-likelihood phylogenetic tree based on 16S rRNA gene sequence comparisons is shown in Figure 1. A Basic Local Alignment Search Tool *(*BLAST) online search at the National Center for Biotechnology Information (NCBI) GenBank was performed for 16S rRNA gene sequences. From the BLAST Search in NCBI GenBank, the HE7 sequence shared 99.85% identity with the 16S rRNA sequence of the strain F2, 93.75% with DOGA 1, 2, 3, 5, 91.66% with *H. marinus* SJ, 89.88% with *H. litoralis* JS5 and 89.43% with *H. vibrionivorans* BL9.

### 2.3. Prey Specificity and Predatory Efficiency of HE7 Against Other MDR E. coli and Salmonella Preys

**Figure 2:**
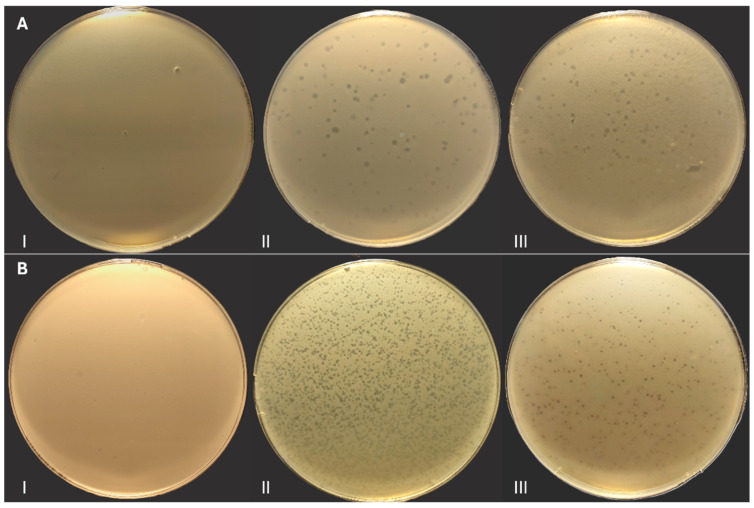
Plaque assay results: (**A I**) negative control (*E. coli)*; (**A II**) lysis plaques on E7; (**A III**) lysis plaques on E3. (**B I**) negative control (*Salmonella)*; (**B II**) lysis plaques on S3; (**B III**) lysis plaques on S9.

HE7 was able to lyse *E. coli* E3 and *Salmonella* S3 and S9 strains. For E3 prey, lysis plaques became visible after 24 h, with a diameter like that of the primary prey, while for both S3 and S9 preys the diameter was smaller, approximately 1 mm (Figure 2).

### 2.4. Challenging HE7/E7

**Figure 3:**
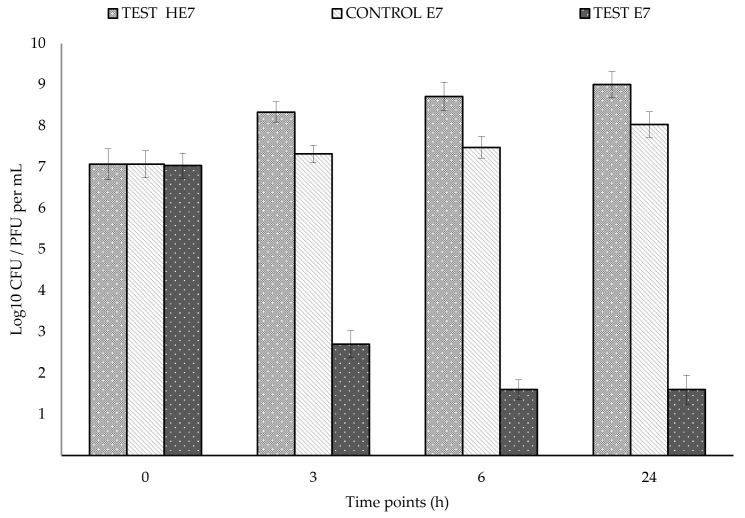
Results of challenge experiment with a ratio of 10^7^ PFU/10^7^ CFU per mL of *Halobacteriovorax* HE7 and *E. coli* E7, respectively, showing the reduction in E7 in the test (with HE7) respect to the control (without HE7) in DNB. t and *p* values for the differences in E7 levels between the test and control: 3 h: t = 6.3542, *p* < 0.0001; 6 h: t = 4.7121, *p* = 0.0008; 24 h: t = 4.5666, *p* = 0.0010.

**Figure 4:**
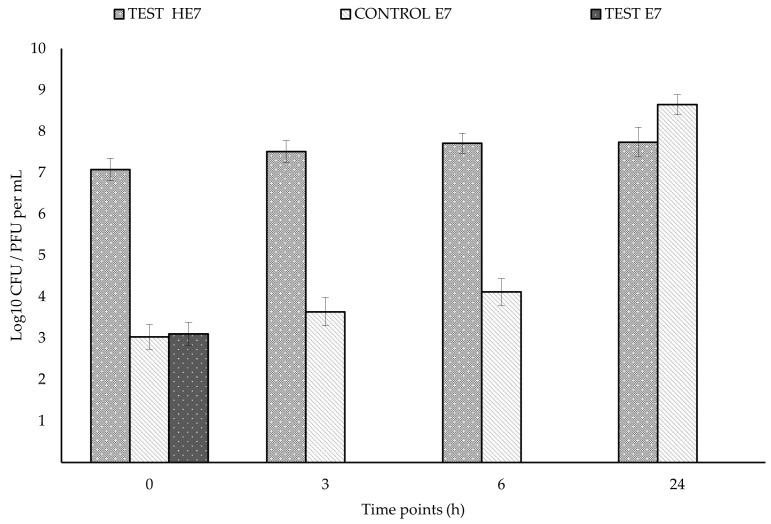
Results of challenge experiment with a ratio of 10^7^ PFU/10^3^ CFU per mL of *Halobacteriovorax* HE7 and *E. coli* E7, respectively, showing the reduction in E7 in the test (with HE7) respect to the control (without HE7) in DNB. t and *p* values for the differences in E7 levels between the test and control: 3 h: t = 3.6909, *p* = 0.0042; 6 h: t = 4.1576, *p* = 0.0020 24 h: t = 4.8674, *p* = 0.0007.

Results of challenge experiment 1 with 10^7^ PFU/10^7^ CFU per mL predator/prey ratio are shown in Figure 3. In the test, the predator increased by 1.3 logs after 3 h and then remained constant. In the test, prey decreased by 5.4 logs between 0 and 6 h, then remained at the same level between 6 and 24 h. In the control, prey progressively increased in 24 h, up to 1.1 × 10^8^ CFU per mL. In the test, a significantly lower level of prey was observed compared to the control from 3 h to 24 h, with a maximum difference of 6.4 logs at 24 h. The results of challenge experiment 2 with 10^7^ PFU/10^3^ CFU per mL predator/prey ratio are shown in Figure 4. In the test, predator concentration increased by 0.7 logs in 24 h. In the test, prey concentration decreased by 3 logs, from 1.3 × 10^3^ to <10 CFU per mL, between 0 and 6 h, then remained at the same level between 6 and 24 h. In control, prey concentration progressively increased in 24 h, up to 4.4 × 10^8^ CFU per mL. In the test, a significantly lower level of prey was observed compared to the control from 3 h to 24 h, with a maximum difference of 8.6 logs at 24 h. 

## 3. Discussion

Due to the growing problem of antimicrobial resistance, there is increasing attention in the scientific community towards alternative approaches to combating AMR pathogens that do not involve the use of antibiotics [3,4,13,27]. The global spread of ESBL-producing *Enterobacteriaceae* represents a major challenge that remains to be solved because it requires the search for new effective therapies against these bacteria [13,27,28]. Predatory bacteria already have well-known applications beyond medicine, such as in the food industry, biocontrol, aquaculture and wastewater treatment [24]. Regarding their potential use in medicine as a therapy for infections by Gram-negative bacteria, their efficacy “in vitro” in eliminating various pathogens, including those resistant to several antibiotics, as well as safety and efficacy “in vivo” in reducing bacterial load in various animal infection models have been demonstrated [9,10,11]. To date, the exploration of predatory bacteria as an alternative to antibiotics has been mostly focused on *B. bacteriovorus* [23,29]. The main problem encountered when using *B. bacteriovorus* as a live antibiotic is that this predator, although effective in containing prey, did not allow their elimination nor a reduction of more than 6 logs [23,30,31]. Previous scientific information has reported that *Halobacteriovorax* is isolated from sea water and preferentially preys on other halophilic bacteria, particularly vibrios [25,32]. Only two previous works [26,33] have reported the isolation of *Halobacteriovorax* using *Enterobacteriaceae* as primary prey from low-salinity water samples. In this work we used as primary prey a CTX-M-producing *E. coli* strain that had been isolated in the Adriatic from bivalve molluscs. The nomenclature and taxonomy of BALOs are continuously updated, and consequently the strains isolated over the years have been classified differently. Regarding the halophilic strains of BALOs, the sequences deposited in databases before 2015 could not be labeled as *Halobacteriovorax*, thus making it difficult to perform a correct taxonomic correlation between them and more recent sequences. Currently, *H. marinus* SJ is the only complete genome of *Halobacteriovoraceae* and draft genomes are available for some other strains characterized by high sequence divergence and relatively low average amino acid identity [34]. It has been hypothesized that horizontal gene transfer also contributes to a rapid evolution of the *Halobacteriovorax* genome [34]. In BALOs, as in other bacteria, a sequence similarity > 98% is required for grouping at the species level [16]. In this study, the sequence similarity of HE7 to the type strains of *Halobacteriovorax* and those isolated from the Adriatic Sea was always <98%, suggesting that HE7 represents a different species than the others. We intend to perform complete genome sequencing of HE7 to obtain more detailed information on its taxonomic position. Salt adaptation makes *Halobacteriovorax* a distinct genus from non-halotolerant BALOs. HE7 demonstrated the ability to grow and predate efficiently at salinity and temperature ranges from 0 to 30 ppt and from 26 to 37 °C, respectively. Despite the fact that HE7 is an environmental strain, it maintained its activity even at 37 °C, although with a lower efficiency than at 26 °C. BALOs are aerobic bacteria and few studies have tested their ability to grow and predate under microaerophilic or oxygen-free conditions [35]. HE7 grew and predated under microaerophilic and anaerobic conditions with an efficiency comparable to that of aerobic conditions. We acknowledge that in this study the influence of physical-chemical parameters on the predation ability of HE7 was preliminarily assessed for a rather short time, i.e., 6 h. This choice was made to avoid the bias of false positive results related to the premature death of prey when grown under suboptimal conditions, especially regarding salt concentration in the medium and anaerobic incubation. These preliminary results on the adaptability of HE7 to different growth conditions make it a worthy candidate for further in vitro investigation of its predatory capacity against AMR bacteria. These preliminary results of HE7 adaptability to different growth conditions encourage further in vitro investigations. Its predation activity in the tested temperature and salinity ranges makes it a good candidate for the biocontrol of AMR bacteria in the food, fishery, aquaculture and agriculture sectors. Its ability to prey in the tested temperature and oxygen tension ranges makes it a good candidate also for clinical use in humans and animals as a therapeutic agent for infections in various body systems, including the intestinal tract, where it may also find application as a probiotic. Regarding this last application, a limitation could be the predator’s ability to tolerate low pH values during transit through the gastric tract. This limitation could, however, be overcome by protecting the predator through encapsulation, which has already been tested with satisfactory results [10]. Although it has already been widely documented that BALOs have no effect on eukaryotic cells [9,10,11], before testing it for clinical applications, cytotoxicity and host safety tests, as well as an evaluation of HE7’s potential influence on the human microbiome, will be necessary. HE7, although captured using a single *E. coli* strain as a primary prey, was able not only to prey on the other tested MDR *Enterobacteriaceae* strains belonging to the same genus, i.e., *E. coli*, but also to prey on a different genus, i.e., *Salmonella,* under the tested experimental conditions. Various predator/prey ratios were preliminarily tested at 6 h over the predator concentration range from 10^3^ to 10^7^ PFU per mL, to select the ratios that exhibited the greatest prey reduction to use for the challenge tests. The most effective HE7 concentration was 10^7^ PFU per mL against the prey at both low (10^3^ CFU per mL) and high (10^7^ CFU per mL) concentrations. In the 10^7^ PFU/10^3^ CFU per mL challenge, HE7 was able to eliminate the primary prey when it was at low concentration already after 3 h, maintaining this effect for at least 24 h. In the 10^7^ PFU/10^7^ CFU per mL challenge, even when the prey was at a high concentration the predator was extremely efficient, with a prey decrease respect to control of about 7 logs already at 6 h. It is our intention to further investigate the bactericidal effect of HE7 by testing other H7/HE7 ratios and other MDR preys, also in longer-term predation experiments. In our previous work with another strain of *Halobacteriovorax* called M7 and isolated with *Salmonella* as primary prey, we observed that to obtain maximum predatory efficiency it had to be used at a high concentration, and this also occurs for HE7 [26]. However, M7, even used at the most effective concentration, was unable to either eliminate prey or to reduce it as effectively as HE7 [26]. This work generally highlights the potential application of *Halobacteriovorax* to be used in the future for the treatment of AMR and MDR pathogens in both non-medical and medical applications. Some peculiar characteristics make *Halobacteriovorax*, and BALOs in general, interesting for the control of AMR bacteria: the absence of AMR genes that induce resistance to predation, the ability to penetrate biofilms and the apparent lack of specific resistance to these predators [10]. However, there are also limitations. The first is the restricted host range, a limitation that could, however, be overcome by using a mix of *Halobacteriovorax* strains active against different prey. Combinations of *B. bacteriovorus* with bacteriophages or antimicrobials, or the use of enzymes derived from predatory bacteria, have already been tested experimentally, with promising results [15,22,24,29]. We also suppose that *Halobacteriovorax* could find application both alone and in an integrated context of antimicrobial strategies alternative to antibiotics. Although it has been reported that BALOs not only kill prey but also degrade the DNA present within them and thus could limit the dispersal of resistance genes [10], the potential transmission of AMR genes and non-specific predation, which could target non-pathogenic commensal bacteria, represent other potential limitations that will require further investigation. The first potential application of HE7 we are currently testing is its use in removing AMR *E. coli* and *Salmonella* from food processing and storage environments. Food, in fact, represents an important link in the chain of transmission of AMR bacteria to humans. These bacteria, originating from the intestines through feces, can reach the marine and terrestrial environments through various routes and thus contaminate food. From food, these bacteria return to humans via the fecal–oral route, and the cycle starts again.

## 4. Materials and Methods

### 4.1. Sampling Site

Two samplings were carried out in July 2024 in estuarine water near the mouth of the Musone river, which is in the south-eastern sector of the Monte Conero Natural Park and flows into the Adriatic Sea between Numana and Porto Recanati. For each sample, two liters of water were collected and placed in sterile polypropylene bottles. A multiparameter probe (Handy Gamma, Oxyguard, Farum, Denmark) was used to measure the water temperature and salinity in situ, which for both samples were 23 °C and 26 ppt, respectively. The samples were immediately transferred to the laboratory under refrigerated conditions and analyzed within 4 h.

### 4.2. Prey Strains

All preys were MDR *E. coli* and *Salmonella* strains belonging to the bacterial collections of the National Reference Laboratory for bacterial contamination of bivalve molluscs and of the Regional Enteropathogenic Centre, of the Istituto Zooprofilattico Sperimentale dell’Umbria e delle Marche (Table 1). The CTX-M-producing *E. coli* strain named E7 (Table 1) was chosen as the primary prey. The *Salmonella* and *E. coli* fresh enrichments were prepared from a stock culture grown on Brain Hearth Infusion broth at 37 °C (BHI Difco; Becton, Dickinson and Company, Milan, Italy) until prey reached an OD600 of 0.8 (~1 × 10^8^ CFU per mL).

### 4.3. Halobacteriovorax Against E7 Detection

The double-layer agar plating technique was used on 500 mL of test water filtered through a 0.45 μm-pore-size Millex-HV syringe filter (Millipore Corp., Billerica, MA, USA) to remove particulates and bacteria [26,32,36,37]. Plaque assays were performed on undiluted and serially diluted 10-fold water in sterilized peptone salt solution. For each assay, 25 mL of modified polypeptone peptone (Pp) plus bacto agar (Pp20; Biolife, Milano, Italy) was dispensed into a 100 mm Petri dish and allowed to harden to make a bottom layer. The top agar was melted and brought to and kept at a temperature of 48 °C in a water bath. The plaque assay was conducted by combining 0.1 mL of primary prey fresh enrichment, 5 mL of filtered undiluted and diluted test water and 10 mL of molten Pp 20 agar (48 °C) in test tubes [32]. The tubes were inverted three times to mix and poured on top of the previously made bottom layer. Plates were incubated at 26 °C after solidification for 5 days. The plaques developed were taken individually and enriched in Diluted Nutrient Broth (DNB; Difco; Becton, Dickinson and Company, Milan, Italy) at 26 °C to obtain pure stock cultures to be used for subsequent tests and for molecular identification by 16S rRNA analysis. Briefly, two- to three-days enrichments were filtered through a 0.45-μm filter to remove prey. The filtrates were serially diluted 10-fold, and the presence of *E. coli* was quantitatively measured by plating on Tryptone Bile X-GLUC (TBX) Agar (Biolife, Milan, Italy) using 100 μL of each dilution, followed by incubation at 44 °C for 24 h to detect the presence of the host. The diluted filtrates were also tested for *Halobacteriovorax* using the double-layer agar plating technique, as previously described. Higher dilutions containing *Halobacteriovorax* but free of *E. coli* were considered pure cultures, stored and used for further investigation [33].

### 4.4. Halobacteriovorax Molecular Identification and Sequencing Analysis

For the PCR assay, single plaques were resuspended in 100 mL of sterile double-distilled water. After shaking at 3000 rpm, the suspension was transferred to a new tube and heated in boiling water for 3 min [25]. Primers Bac676F and Bac1442R were used for analysis on a fragment of the 16S rRNA gene, as previously described [19,20]. Isolates showing a 700 bp band were identified as *Halobacteriovorax* [19,20] considering that they came from estuarine water containing 26 ppt of NaCl. Sequencing analysis was performed on a 700 bp PCR product from a single plaque for each positive sample using the BAC1442R primers and the ABI Prism^®^ BigDye^®^ Terminator v1.1 Cycle Sequencing Kit (Applied Biosystems^TM^, Life Technologies, Foster City, CA, USA) [19]. The High Pure PCR Product Purification Kit from Roche Diagnostics (GmbH, Mannheim, Germany) was used to purify the PCR products. The ABI Prism^®^ 310 Genetic Analyzer automated capillary sequencer (Applied Biosystems^TM^, USA) was used for analysis of the sequenced products. Manually edited and aligned nucleotide sequences were analyzed with CLC genomics workbench V.12 (Qiagen Bioinformatics, Hilden, Germany). By conducting a BLAST search in NCBI GenBank, the HE7 sequence was compared with those of the following type strains of *Halobacteriovorax*: *H. marinus* SJ (GenBank accession number NR102485), *H. litoralis* JS5 (GenBank accession number NR028724) and *H. vibrionivorans* BL9 (GenBank accession number MH150810). Moreover, the HE7 sequence was compared to those of another four *Halobacteriovorax* strains that we had previously isolated in Adriatic Sea named DOGA 1,2,3,5 (GenBank accession numbers MN750616, MN750617 MN750618, MN750620 [25]. Finally, the HE7 sequence was compared with the sequence of the taxonomically closest strain, named F2 (GenBank accession number AY294218). Phylogenetic trees of the 16S rRNA gene sequences were reconstructed using the maximum likelihood (ML) method. The software applied was the Mega-6 software [38,39]. A Maximum Likelihood Tree was created using the Tamura-Nei model to estimate the genetic distance of nucleotide sequences and bootstrap method 1000.

### 4.5. Prey Specificity and Predatory Efficiency of Halobacteriovorax Against Other AMR E. coli and Salmonella Preys

For the specificity test, the fresh enrichment of *Halobacteriovorax* (concentration of about 10^8^ CFU per mL) was prepared by combining DNB 1 mL of the predator stock cultures and 0.1 mL of the primary prey fresh enrichment prepared from a stock culture. After incubation at 26 °C for 24 h, the fresh enrichments of *Halobacteriovorax* were filtered through a 0.45 μm filter to remove the primary prey. Prey specificity and predator efficiency of *Halobacteriovorax* strains were determined by monitoring their abilities to form clear lytic halos on a lawn of AMR prey listed in Table 1, using a double-layer agar plating technique on a layer of prey at 26 °C [32,36,37]. Briefly, 1 mL of filtered predator undiluted fresh enrichment and 0.1 mL of prey fresh enrichment were added to 10 mL of molted Pp 20 top agar in tubes. The tubes were inverted three times to mix and poured on top of the earlier prepared bottom layer and then incubated after hardening. For each prey specificity assay a positive control, represented by primary prey and the *Halobacteriovorax* strain, and a negative control, which consisted of the prey alone, were performed.

### 4.6. Preliminary Tests to Define the Most Effective Predator/Prey Ratio on Prey Reduction and the Optimal Physical-Chemical Parameters to Be Used in Challenge Experiments

Different predator/prey concentrations were tested, based on our previous studies [25,26,32], to obtain data on predator activity in the predator range from 10^3^ to 10^7^ PFU per mL. Test flasks with 10 mL DNB were inoculated with the following predator/prey concentrations, 10^7^ PFU/10^7^ CFU per mL, 10^7^ PFU/10^3^ CFU per mL, 10^6^ PFU/10^6^ CFU per mL, 10^6^ PFU/10^3^ CFU per mL, 10^5^ PFU/10^5^ CFU per mL, 10^5^ PFU/10^3^ CFU per mL, 10^4^ PFU/10^4^ CFU per mL and 10^3^ PFU/10^3^ CFU per mL, to define the most effective predator/prey ratio. The same prey concentrations were inoculated into DNB without the predator, as a control for each test microcosm. To evaluate predator–prey interactions, three formulations of DNB containing 0, 26 and 30 ppt NaCl were tested. The 26 ppt salt concentration was chosen because it was the optimal concentration, like that of the environment from which it was isolated. The 0 and 30 ppt salt concentrations were used to assess whether it could also prey in non-halophilic and/or more halophilic environments. Moreover, each challenge test was performed at 26 °C, 30 °C and 37 °C under aerobic, microaerophilic and anaerobic conditions. The anaerobic atmosphere was reproduced using pouches AnaeroGen (Thermo Scientific, Waltham, MA, USA), whereas microaerophilic conditions were reproduced using CampyGen pouches (Thermo Scientific, Waltham, MA, USA). For each challenge, the incubation was 6 h long. At 0 and 6 h, prey counts were performed in the test and control microcosms. To count the prey, 1 mL of DNB 10-fold serial dilutions in peptone salt solution were inoculated on Tryptone Bile X-GLUC (TBX) Agar (Biolife, Milan, Italy) and the plates were incubated at 44 °C ± 1 °C for 24 h. Each experiment was replicated twice. Between duplicate experiments, the difference in prey counts had to remain within 0.5 logarithms (logs), as this was the repeatability limit determined by our previous studies [24]. Counts were averaged and log-transformed, and then the log difference between test and control counts were calculated for each predator–prey ratio. The difference in prey counts from duplicate experiments always fell within the defined repeatability limits. After 6 h, prey counts in the controls of the different tests were similar, demonstrating that at least in the first 6 h the different physical-chemical conditions did not influence their growth. The highest prey reductions were reached with 10^7^ PFU/10^7^ CFU and 10^7^ PFU/10^3^ CFU per mL predator/prey ratio in DNB incubated at 26 °C and 30 °C, regardless of salt concentration (0, 26, 30 ppt) and different oxygen conditions in the incubation (Table 2).

### 4.7. Challenging Halobacteriovorax/E7

Two challenge experiments were performed at 26 °C in DNB without NaCl and in aerobic conditions using 10^7^ PFU/10^7^ CFU and 10^7^ PFU/10^3^ CFU per mL predator/prey ratios, which had achieved the greatest prey reduction at the preliminary tests. Before each experiment, predator and prey were counted from the fresh enrichments using double-layer agar plating and pour plate techniques, respectively, obtaining the expected results. In the first experiment, 10 mL of DNB containing predator and prey were tested at concentrations of 10^7^ PFU/10^7^ CFU per mL, respectively. In the second experiment, 10 mL of DNB containing predator and prey were tested at concentrations of 10^7^ PFU/10^3^ CFU per mL, respectively. For both the experiments, 10 mL of DNB without predator containing similar concentrations of prey was tested as a control. Cultures were incubated at 26 °C, monitoring the prey via bacterial counts on plates and the predator via the double-layer agar plating technique, both at 0, 3, 6 and 24 h [21,24].

### 4.8. Statistical Analysis

Both challenges were performed in three separate trials, each in duplicate (*n* = 6). Microbiological counts were reported as mean values (log-transformed) ± standard deviation. Student’s *t*-test (t) with a probability of error (*p*) < 0.05 using a Graph Pad *t*-test calculator was used to assess significant differences in predator and prey counts.

## 5. Conclusions

As far as we are concerned, this is the first work testing in vitro the efficacy of *Halobacteriovorax* against AMR pathogens. Our results demonstrate that HE7 is a potential biocontrol agent against AMR *E. coli* and *Salmonella* and could represent a valid alternative to conventional antimicrobials. Further in vitro and in vivo studies on the predator’s biological properties, its efficacy under different experimental conditions and against various AMR prey and its potential adverse effects on the host will be necessary to move towards practical application of HE7 in both non-medical and medical settings.

## Figures and Tables

**Table 1:** MDR *E. coli* and *Salmonella* preys tested with *Halobacteriovorax*.

Laboratory Identification	Species	Origin	MDR Patterns
E7 (primary prey)	*E. coli*	*Chamelea gallina*	ESBL *bla*CTX-M-55 AMP FOT CIP CHL NAL TMP TET SMX FEP
E3	*E. coli*	*Chamelea gallina*	ESBL *bla*CTX-M-1 AMP FOT TMP TET SMX FEP
S3	*Salmonella Infantis*	Human urine	ESBL *bla*CTX-M-1 AMP FOT KAN NAL TET SMX SXT
S9	*Salmonella Havana*	Ring test	AmpC-phenotype FOX TAZ

Ampicillin (AMP), Cefepime (FEP), Cefotaxime (FOT), Ceftadizime (TAZ), Cefoxitin (FOX), Gentamicin (GEN), Streptomycin (STR), Kanamycin (KAN), Nalidixic acid (NAL), Ciprofloxacin (CIP), Trimethoprim (TMP), Sulfamethoxazole (SMX), Trimethoprim-sulfamethoxazole (SXT), Tetracycline (TET), Chloramphenicol (CHL), Azithromycin (AZI).

**Table 2:** *E. coli* E7 reduction after 6 h of exposure to *Halobacteriovorax* HE7.

PFU Predator/CFU Prey per mL	Log Prey Reduction in Test Respect to Control	
	26–30 °C 0–30 ppt aer/ma/ana Conditions	37 °C 0–30 ppt aer/ma/ana Conditions
10^7^/10^7^	6	4
10^7^/10^3^	4	2
10^6^/10^6^	3.5	2
10^6^/10^3^	3	2
10^5^/10^5^	1	0.5
10^5^/10^3^	1	1
10^4^/10^4^	1	1
10^3^/10^3^	1	0.3

aerobic (aer), microaerophilic (ma), anaerobic (ana).

## Data Availability

The original contributions presented in this study are included in the article. Further inquiries can be directed to the corresponding authors.

## Abbreviations

The following abbreviations are used in this manuscript:

AMC	Antimicrobial-Resistant
BALOs	Bdellovibrio And Like Organisms
CFU	Colony Forming Unit
CTX-M	*bla*CTX-M genes
DNB	Diluted Nutrient Broth
ESBL	Extended Spectrum β Lactamase
PFU	Plaque Forming Unit
Pp	Polypeptone peptone
TBX	Tryptone Bile X-GLUC Agar
